# Improving the Compliance of Intraoperative Antibiotic Redosing: A Quality Improvement Initiative

**DOI:** 10.1097/pq9.0000000000000285

**Published:** 2020-04-10

**Authors:** Michelle M. LeRiger, Amber R. Phipps, Bridget M. Norton, Rachel A. Spitznagel

**Affiliations:** From the * Department of Anesthesiology, University of Nebraska Medical Center, Omaha, Neb.; † Division of Pediatric Anesthesiology, Children’s Hospital & Medical Center, Omaha, Neb.; ‡ VP Quality and Patient Safety, Children’s Hospital & Medical Center, Omaha, Neb.; § Department of Pediatrics, University of Nebraska Medical Center, Omaha, Neb.; ¶ Division of Pediatric Critical Care Medicine, Children’s Hospital & Medical Center, Omaha, Neb.

## Abstract

**Introduction:**

At Children’s Hospital and Medical Center in Omaha, Nebraska, the intraoperative antibiotic redosing guidelines and the time frame considered compliant for redosing were unclear. This lack of clarity plus an ill-defined process for ensuring intraoperative antibiotic redosing resulted in a compliance rate of 11%. The organization’s surgical site infection (SSI) rate was 3.19%, above the national benchmark of 1.87%. The primary project goal was to increase intraoperative antibiotic redosing compliance. The secondary project goal was to decrease SSIs.

**Methods:**

With recommendations from the Infectious Disease Society of America, we developed new organizational redosing guidelines, as well as a new antibiotic-specific reminder alert in the electronic medical record. Implementation of the new guidelines and processes occurred after providing education to the anesthesiologists, surgeons, and circulating nurses. Monthly evaluation of data allowed for quick recognition of oversights followed by the initiation of process updates.

**Results:**

Data showed that the initial compliance rate for the intraoperative redosing of antibiotics was 11%. Following interventions, compliance has reached and sustained an average of 99%. Survey results show that provider knowledge of the guidelines and process has improved. Though not directly related, the National Surgical Quality Improvement Program observed that the SSI rate decreased from 3.19% in 2014 to 2.3% in 2018.

**Conclusions:**

This project demonstrates that comprehensive education along with antibiotic-specific electronic medical record alerts significantly increased the compliance of intraoperative antibiotic redosing at Children’s Hospital & Medical Center. Continuous education and monthly updates sustained results for over 40 months.

## INTRODUCTION

Surgical site infections (SSIs) are infections of the incision or organ space that occur after surgery.^1,2^ SSIs are reported in approximately 2%−3% of surgical procedures and are associated with increased morbidity and mortality.^1–3^ The cost of treating SSIs in the United States is increasing.^1^ Solutions for Patient Safety (SPS) estimates an average cost of $27,000 per SSI, with a range of $11,778 to $42,177.^4–6^ The cost can exceed $90,000 per SSI when an antimicrobial-resistant organism is involved.^1^ Annually, the estimated cost to the U.S. health system is between 15.8 and 57.2 billion dollars.^2^ It is often difficult to determine the cost of an individual SSI, and this is true at Children’s Hospital & Medical Center because direct cost accounting is not used. Approximately 40% to 60% of SSIs are preventable.^7^ Correctly administered perioperative prophylactic antibiotics decrease the rate of SSIs.^1,2^ Clinical practice guidelines for antimicrobial prophylaxis published in the *American Journal of Health-System Pharmacy* recommend intraoperative antibiotic dosing intervals during surgical procedures based on the pharmacokinetics of each drug.^2,8^ In the cardiac surgical patient population, correct intraoperative antibiotic redosing has decreased SSI rates.^3^

At the Children’s Hospital & Medical Center in Omaha, Nebraska, the SSI rate was higher than the national benchmark based on data from the National Surgical Quality Improvement Program (NSQIP) database. NSQIP uses a systematic sampling process to ensure a representative sample of surgical cases. It excludes cardiac, ophthalmology, interventional radiology, dental procedures, and endoscopies. The hospital contributes to the NSQIP database entering 13% of cases annually. In 2014, Children’s SSI rate for all cases was 3.19%, whereas the benchmark was 1.88%. The NSQIP database identified 42 SSIs. Therefore, based on the hospital’s total surgical volume, approximately 315 SSIs occurred in 2014.

Our hospital created an SSI workgroup to implement policies based on SPS recommendations to decrease SSIs. SPS is a network of over 130 children’s hospitals with the shared goal of harm reduction by designing and advising on the implementation of evidence-based bundles to reduce the harm caused by hospital-acquired conditions and serious safety events.^9^ SSIs are the fourth largest contributor to harm reported across the SPS network.^9^ SPS designed a bundle to reduce harm from SSIs to hospitalized children.^9^ Bundle elements include preoperative baths, no razor use, appropriate preoperative antibiotic timing, appropriate skin antisepsis, and appropriate antibiotic redosing (Table 1).^9^

**Table 1.**
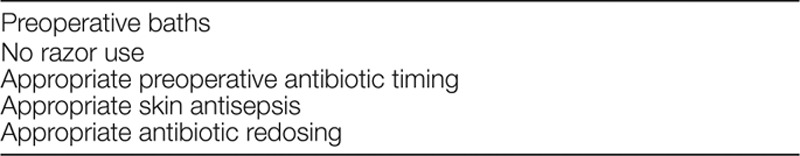
Solutions for Patient Safety Bundle Elements for Prevention of Surgical Site Infections

The primary goal of this Quality Improvement (QI) project was to increase intraoperative antibiotic redosing compliance from 11.2% to 100% for qualifying cases. As referenced, the time frame for this project aligned with our hospital’s focus on improving patient safety and decreasing hospital-acquired conditions. Therefore, a secondary project goal was to decrease SSIs.

## METHODS

We identified a need to establish consistent intraoperative antibiotic redosing guidelines and define the exact time frame, in minutes, considered compliant for intraoperative antibiotic redosing. Additionally, a well-defined process for ordering intraoperative antibiotics was necessary. QI projects are exempt from institutional review board application and approval. This project utilized the Plan Do Check Act (PDCA) methodology (Table 2), thorough data analysis, and on-going education.^10^

**Table 2.**
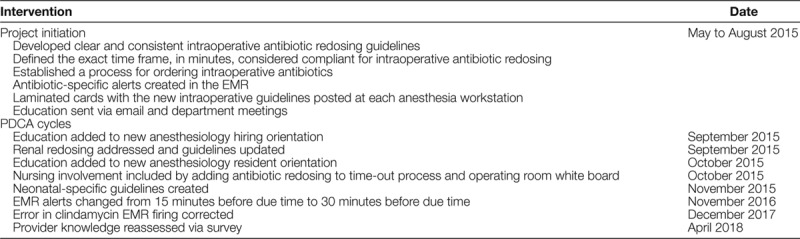
Intraoperative Antibiotic Redosing Project Timeline

Our project team consisted of 2 anesthesiologists, a surgeon, an operating room (OR) nurse, a pharmacist, and an information technology (IT) specialist. In May 2015, we gathered baseline data. Concurrently, monthly meetings were held with the SSI workgroup to review current processes. The SSI workgroup consisted of a surgeon from each subspecialty, a pharmacist from the Antimicrobial Stewardship Program, members from the Performance Improvement Department, an IT specialist, OR nursing managers, and 2 anesthesiologists. At these meetings, research into existing intraoperative antibiotic redosing guidelines and policies took place. Most committee members, including the anesthesiologists, surgeons, and OR nurses, were unaware of formal antibiotic redosing guidelines. However, we discovered that hospital guidelines did exist via a paper ordering sheet. In June 2015, members of the project team worked with members of the hospital’s Antimicrobial Stewardship Program to establish perioperative antimicrobial guidelines utilizing information from the Infectious Disease Society of America and information compiled from hospitals that are part of the Children’s Hospital Association Network (Table 3).^8,11,12^ This work outlined procedures and drug-specific timeframes for intraoperative antibiotic redosing. To be deemed compliant with the redosing, antibiotics needed to be administered 30 minutes before or after the electronic medical record (EMR) redosing due time, determined by the antibiotic administration start time. If a procedure was longer than 2 half-lives, the drug was to be readministered. Renal function did not play a specific role in the creation of the guidelines; however, the pharmacy looks at the creatinine clearance if available on each patient before fulfilling a requested redose.

**Table 3.**
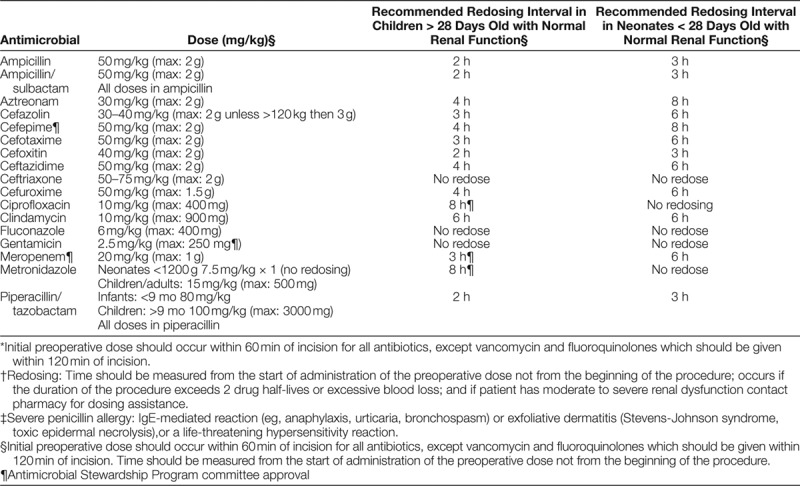
Perioperative Antimicrobial Guidelines*†‡

**Fig. 1.**
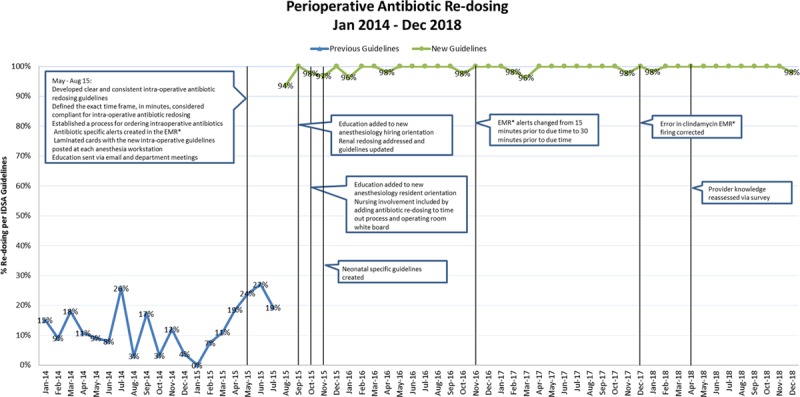
Perioperative antibiotic redosing compliance. This run chart displays the compliance rates of intraoperative antibiotic redosing for all qualifying cases. The team analyzed 2014 and early 2015 data using the preexisting guidelines. Project initiation was August 10, 2015, after which the team analyzed data using the new guidelines. Important PDCA cycles are identified. IDSA, Infectious Disease Society of America.

With new guidelines established, the project team collected baseline data from all qualifying intraoperative cases. This analysis excluded antibiotics given outside of the OR. Initial data analysis was calculated using the antibiotic-specific redosing time frames from the preexisting guidelines. These guidelines required antibiotic redoses to be given within a 5-minute window on either side of the due time. If a case required more than 1 redose, all required redoses needed to be administered correctly, or the case was considered noncompliant. As stated, most physicians were unaware of these guidelines. In June 2015, we assessed surgeon and anesthesia provider knowledge using a survey. Of the 48 who received the survey, 20 responded for a response rate of 41.7%. Only 41% of responders felt that they had a clear understanding of the redosing guidelines. The lack of clarity is evident by the average 2014 compliance rate of 11.2% (Fig. 1).

Following the development of guidelines and the collection of data, the project team worked with IT analysts to create intraoperative antibiotic reminder alerts for anesthesia providers. An antibiotic-specific redosing reminder was created in the federally qualified EMR by August 2015. The alert appears on the left side of the anesthesia record, flashing yellow 15 minutes before the antibiotic is due. It changes to red when the antibiotic becomes due. The project team created laminated cards with the new guidelines and posted them at every anesthesia workstation. Education on the new guidelines, the time frame considered compliant for redosing, and the new EMR alert was provided at department meetings in August 2015 and sent via email to all anesthesiologists and surgeons. The process for ordering the antibiotics consisted of the OR circulating nurse calling the pharmacy to request a redose. All education was provided before the project go-live date of August 10, 2015. Newly hired anesthesiologists received the information regarding the guidelines and processes in their orientation.

Following the project initiation, monthly evaluation of EMR data occurred. The Performance Improvement Department conducted an initial data review with potential cases of noncompliance sent to the anesthesiologists on the project team. The review of these noncompliant cases involved peer-to-peer conversations and revisions in the process followed by further education if required (Table 2). Anesthesiologists often notated in the record as to why the antibiotic redose was late or missed. This notation was crucial as sometimes the peer-to-peer conversations would take place 3 weeks after surgery, making recall difficult. By analyzing noncompliance, we identified oversights in the process, recurring issues with providers, and issues at specific locations or times of day (Table 2). Anesthesia providers received monthly updates on compliance rates. In October 2015, the anesthesia resident involved in a missed antibiotic redose was unaware of the hospital’s new guidelines and thought the EMR alert was incorrect and therefore ignored it. Education sessions or emails about the process and guidelines excluded anesthesia residents as identified by the missed antibiotic dose. Anesthesia residents rotate through the ORs monthly and play an important role in direct patient care. To address this oversight, we included the Perioperative Antimicrobial Guidelines and education on the EMR alerts in the resident orientation. This oversight also highlighted the problem that redosing was strictly reliant on the anesthesia team. Therefore, the time-out process was updated to include a discussion on antibiotic redosing. The circulating nurse writes the redosing time on the surgical whiteboard for all members of the OR team to see, adding a layer of protection in the process. In November 2015, a provider recognized that the new guidelines did not address redosing in neonates. Team leaders discussed this with the team pharmacist, who created separate neonatal guidelines for those less than 28 days of age with normal renal function. In November 2016, during data analysis and with input from anesthesiologists, it was determined that misses or near misses were occurring off hours (after 5 pm and on the weekends) and in non-OR locations (such as the fetal care center). To account for this, we updated the EMR antibiotic reminder to alert 30 minutes before the time due, allowing staff more time to order and physically receive the antibiotics. Project leaders discussed these obstacles at group meetings and encouraged anesthesia providers to order the antibiotics early in these circumstances. The pharmacy representative on the team was also made aware of this problem to provide education to the pharmacy staff. Providers received updates when new PDCA cycles or updates in the process occurred.

The framework for the composition of this article utilized the Standards for QUality Improvement Reporting Excellence (SQUIRE) guidelines.

## RESULTS

Following project implementation, compliance with intraoperative antibiotic redosing increased from the 2014 average of 11.2% to 89% within the first month. Analysis of August data started on the implementation date of August 10. At the end of the second month, compliance rates had increased to 100% (Fig. 1). Data evaluation continues monthly, with compliance averaging 99.2% over the last 41 months. The team implemented updates, focused on education of changes, and stressed the importance of communication when they discovered potential gaps in the process (Table 2). Although not all of those changes increased compliance rates, the changes needed to occur to prevent future errors.

In April 2018, anesthesia providers were resurveyed using the same set of questions. Seventy-five percent of anesthesia providers who responded had a clear understanding of the frequency at which commonly used antibiotics should be redosed intraoperatively according to the hospital guidelines, a 33% improvement. Based on new survey results, anesthesia provider knowledge regarding the correct redosing timeframe considered complaint was also 75%, an improvement of 50% from project initiation.

The antibiotic redosing team and hospital’s SSI workgroup worked well together. The SSI workgroup’s goal was to institute the bundle modeled off of the SPS bundle to decrease the rate of SSIs (Table 1). In January 2016, we implemented the final element of the bundle. As mentioned, one part of this bundle was appropriate antibiotic redosing. Specific data on the compliance rates of other portions of the bundle were not available due to inconsistent record keeping. At project initiation, our hospital’s SSI rate was 3.19% for 2014, when the overall rate in the NSQIP database was 1.88%. Our hospital’s SSI rate has decreased to 2.09% as of January 2018, which correlated to the observed rate of SSIs for the entire NSQIP database.

## DISCUSSION

This QI initiative defined evidence-based intraoperative antibiotic redosing guidelines and the timeframe considered compliant for antibiotic redosing. It utilized an antibiotic-specific EMR alert to remind anesthesia providers when an antibiotic was due. Throughout the project, monthly evaluation of data allowed for quick recognition of oversights or problems followed by the initiation of process updates. Although not all PDCA cycles or revisions resulted in noticeable change, they did ensure that high compliance rates were sustained.

The use of EMR alerts as part of single or multifaceted improvement initiatives has been reported in other studies.^2,3,7^ One university-associated hospital incorporated EMR reminders as part of a multifaceted approach to improving intraoperative antibiotic redosing. Their compliance rates improved by 15% to approximately 80%.^2^ Another study looking only at the use of EMR alerts to improve the administration of the second antibiotic dose found an absolute improvement of 17.1%, with approximately 70% compliance rate.^7^ This study showed sustained compliance rates 7 months later. Our compliance rates increased the first month significantly (Fig. 1) and have remained high for over 41 months, averaging 99.2%. We believe that our compliance rates rose so significantly because our institution’s previous guidelines were unclear and that our compliance rates are higher than those reported by other institutions because of the multilayered approach of our study. We utilized preimplementation education, and to sustain momentum, we sent reports of monthly audits and project updates to anesthesia providers via email and communicated at department meetings.

Additionally, providers involved in noncompliance would get an individual email as a reminder to maintain vigilance. By evaluating all possible cases of noncompliance for accuracy and any notes made by the anesthesia providers, we were able to implement changes to ensure sustainability. Combining extensive education with the EMR antibiotic-specific reminders highlights the strengths of this project.

Although timely notification in the EMR is likely the most important factor in achieving compliance, several other factors likely contributed. The new guidelines were widely disseminated and accessible, whereas the previous guidelines were not. Additionally, the new guidelines were updated to reflect the definition of compliance used throughout the rest of the institution, which states that redoses should be administered within a 30-minute window on either side of the due time. Analysis of baseline data using the preexisting guidelines required a stricter 5-minute redosing window to be compliant.

One limitation of the project is that antibiotic ordering still requires diligence on the part of the OR team and, ultimately, the anesthesia providers. Upon initial development of the reordering process, the project team wanted the EMR to alert the OR pharmacy when an antibiotic redose was near due; however, limitations in the EMR made this impossible, and we were unable to identify an alternative solution for pharmacy involvement in the reordering process. Per hospital policy, physicians order all antibiotics from the pharmacy to ensure administration of the correct dose, which can take additional time. And, as with any QI project, communication is imperative. Some providers remained unaware of the changes implemented with this project. The education initially excluded residents and new providers, as we identified with monthly audits. An additional limitation is that OR nurses do not always write the antibiotic redosing time on the whiteboard despite this being part of the time-out process. We have not initiated any PDCA cycles to improve this, but it is something we could consider in the future. Finally, we did not have a control group without intervention because the project included all cases meeting requirements for redosing by nature of the QI initiative.

Due to the shortened redosing interval of some antibiotics, there is a concern for acute kidney injury, so in September 2015, the team worked on updating the guidelines to address renal dosing. To our knowledge, there have not been any cases of acute kidney injury reported.

Improved compliance with intraoperative antibiotic redosing was one component of the bundle initiative instituted by the hospital’s SSI workgroup. We cannot delineate the impact of the correct antibiotic redosing on the overall decrease in the SSIs. Although the antibiotic redosing portion of the SSI bundle has remained consistent, data on the compliance rate of all portions of the bundle are not available due to inconsistent record keeping.

Although we cannot draw a clear correlation between the SSI rate and our improved intraoperative compliance redosing, correct intraoperative antibiotic redosing has been shown to decrease SSIs,^3^ and this QI project demonstrates that comprehensive education along with antibiotic-specific EMR alerts significantly increased the compliance of intraoperative antibiotic redosing. Through effort by many individuals, the changes have now become standard practice. Utilizing continual education and monthly updates, we have sustained results for over 40 months.

## DISCLOSURE

The authors have no financial interest to declare in relation to the content of this article.

## ACKNOWLEDGMENTS

The authors would like to acknowledge the Children’s Hospital & Medical Center Performance Improvement Department, IT Department, Pharmacy, and Antimicrobial Stewardship Program for their contributions and assistance throughout the project.
